# Sudden Headache and Blindness Due to Pituitary (Adenoma) Infarction: A Case Report

**DOI:** 10.7759/cureus.4059

**Published:** 2019-02-13

**Authors:** Graham Dupont, Stefan Lachkar, Joe Iwanaga, R. Shane Tubbs, Basem Ishak

**Affiliations:** 1 Neurosurgery, Seattle Science Foundation, Seattle, USA; 2 Anatomy, Seattle Science Foundation, Seattle, USA; 3 Medical Education and Simulation, Seattle Science Foundation, Seattle, USA

**Keywords:** pituitary infarction, sudden blindness, opthalmoplegia, pituitary failure

## Abstract

Pituitary infarction (PI) is a rare medical emergency appearing in patients with pituitary adenoma, presenting with sudden onset of severe headache, and often associated with vomiting, nausea, visual deterioration, and decreased consciousness. We describe an 83-year-old woman who presented with blindness after a history of severe headache. An urgent computed tomography (CT) scan of the brain had been arranged, and a massive pituitary adenoma with hemorrhage was observed, and admission to the neurosurgical department followed. A multidisciplinary team comprising a neurosurgeon, an ophthalmologist, and an endocrinologist is required to manage such cases. To confirm the diagnosis, an urgent magnetic resonance imaging (MRI) must be performed; CT scan can be indicated if MRI is contraindicated or not possible.

## Introduction

Pituitary infarction (PI), also known as pituitary apoplexy, is a potentially life-threatening condition due to ischemia or acute hemorrhage of the pituitary gland, often occurring as a result of invasive pituitary adenoma resection [1-2]. Patients present with sudden headache, nausea, vomiting, features of meningism, visual deterioration, ophthalmoplegia, and partial or complete pituitary failure. The rapid aggravation of vision is a critical condition as the tolerance of the optic nerves to permanent damage is unforeseeable [3]. The application of corticosteroids has dramatically reduced the mortality of PI, and more recently, multidisciplinary teams and highly equipped medical centers make PI far more manageable. The clinical course varies tremendously from asymptomatic to critical patients with loss of vision and subarachnoid hemorrhage. Therefore, PI is often misdiagnosed, and optimal management remains controversial due to complications following surgical resection of pituitary neoplasms such as transient diabetes insipidus, cerebrospinal fluid (CSF) leak, delayed hypopituitarism, posterior reversible encephalopathy syndrome (PRES), and permanent diabetes insipidus [4-5]. Furthermore, fluctuations in blood pressure or vasospasms due to surgical positioning and duration may also result in postsurgical hemorrhage of existing pituitary adenomas [6]. Herein, we report a case of an 83-year-old woman who presented with bilateral blindness.

## Case presentation

**Figure 1:**
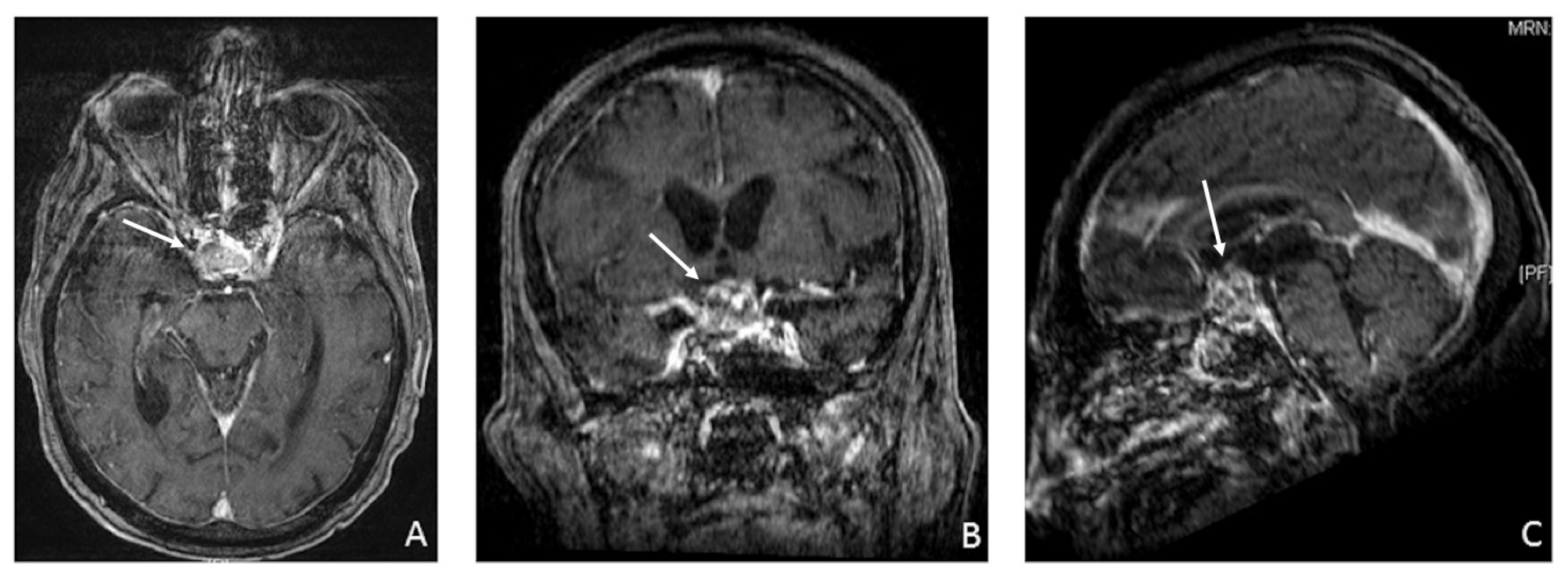
Gadolinium-enhanced T1-weighted magnetic resonance imaging (MRI) scans demonstrating a large pituitary macroadenoma with hemorrhage. Histology revealed pituitary (adenoma) infarction compressing the optic chiasm (white arrow). Axial (A), coronal (B), and sagittal (C) views of the brain.

The 83-year-old patient presented herself at a neurological emergency department with bilateral blindness and a history of severe headache, which she described the day before. An urgent computed tomography (CT) scan of the brain had been arranged, and a massive pituitary adenoma with hemorrhage was observed, and admission to the neurosurgical department immediately followed. When arriving at the hospital the hemodynamic parameters were stable (BP 130/90, 65 BPM); Glasgow coma score was 14/15. Her medical history included chronic renal failure (III°) as well as cardiovascular disease with hypertension, chronic atrial fibrillation, coronary heart disease with percutaneous transluminal coronary angioplasty, and stent. Therefore, dual oral anticoagulation with clopidogrel and acetylsalicylic acid was administered. Multiple electrode aggregometry did not detect effects of both anticoagulants. The neuro-ophthalmic examination revealed no perception of light in both eyes. Fundoscopy was normal without pallor of the optic nerves, no meningeal signs, and no overt clinical signs of hormone imbalance, but the measured pituitary hormone profile had a slight elevated prolactin level and suppression of sex hormones and thyroid profile (Table 1). The blood tests revealed normal electrolytes and infection parameters. A magnetic resonance imaging (MRI) demonstrated a large 22 mm x 18 mm x 15 mm enhancing hemorrhagic pituitary macroadenoma with suprasellar extension and compression of the optic chiasm (Figure 1). Emergency surgery was indicated. A transsphenoidal approach was carried out under general anesthesia. The pituitary tumor was identified, though it was not characteristic of a hemorrhagic pituitary macroadenoma. The tumor was tough and yellow colored, and debulking with a sharp curettage was difficult. Acute hemorrhage ceased after resection of tumor. Dopamine agonist was not used due to the patient experiencing complete blindness. After decompression, the surgery was concluded. After a period of cardio-pulmonary stability in the ICU, the patient was extubated. In this case, the transsphenoidal decompression of the optic chiasm provided an improvement in both eyes. Her visual disturbance improved with visual acuity from 0 to 5/100 for both eyes postoperatively. Counting fingers, identifying persons, and color vision were possible. On the first postoperative day, she was anxious and complained of gradual increase in shortness of breath. A non-ST elevation myocardial infarction was diagnosed. Additionally, to that, she developed an acute renal failure requiring temporary hemodialysis. After a short period, kidney function recovered, and cardiovascular stability was observed. The pathological examination confirmed a pituitary adenoma, most of it undergoing infarction. 

**Table 1:** Pituitary hormone profile of patient with sudden acute blindness from pituitary adenoma. mU/l = milliunits per liter; U/l = units per liter; ng/l = nanogram per liter.

Pituitary axis hormone	Measured level (reference range)
Thyroid stimulating hormone	0.2 mU/l (0.4–4.0)
Free thyroxine (fT4)	8.4 ng/l (8–18)
Prolactin	713 mU/l (60–620)
Follicle stimulating hormone	2.3 U/l (18–153)
Luteinizing hormone	1.7 U/l (16–64)

## Discussion

TThe first clinical case of PI was described by Percival Bailey in 1896 [7]. PI is a rare medical emergency appearing in patients with pituitary adenoma, presenting with sudden onset of severe headache, often associated with vomiting, nausea, visual deterioration, and decreased consciousness. Secondary renal failure is also common in patients following PI [8]. Misdiagnosis remains common due to diagnostic difficulties, which can result in significant morbidity [9]. This aspect leads to confusion and delay in management that may be problematic in the future for the patient. If diagnosed, patients should be referred to a multidisciplinary team comprising a neurosurgeon, an ophthalmologist, and an endocrinologist; conservative management versus neurosurgical intervention should be always considered [8]. Lack of expertise in local hospitals is still a problem. Patients should be managed in a center with neurosurgery, endocrinology, and ophthalmology expertise on-site. To confirm the diagnosis an urgent MRI must be done. A CT scan can be indicated if MRI is contraindicated or not possible [8]. PI can also occur in other specific diseases, e.g., Sheehan’s syndrome, caused by ischemic necrosis due to blood loss and hypovolemic shock during and after childbirth. Other factors such as radiation therapy, head trauma, coagulopathies, anticoagulation therapy, systemic hypertension and major surgery, and especially coronary artery bypass grafting have also been identified to precipitate PI [10-12]. Concerning the international literature, there is certainly a lack of randomized studies. The 2010 published United Kingdom (UK) national guidelines contain recommendations related to the clinical, endocrinological, and radiological assessments and management to improve the treatment of this rare but potentially life-threatening condition [9]. Urgent surgical decompression of the pituitary gland is required in patients with severe neuro-ophthalmic deficit resulting in blindness [13], yet the role of urgent decompression in patients with a mild symptomatic or without neurological deficit remains controversial, as the physiology of PI is still rather unclear [14]. Nevertheless, a multidisciplinary team is vital for decisions regarding PI that may precipitate as fatal.

## Conclusions

Urgent surgical decompression of the pituitary gland is required in patients with severe neuro-ophthalmic deficit resulting in blindness, yet the role of urgent decompression in patients with a mild symptomatic or without neurological deficit remains controversial.
